# Shifts in seasonal timing of respiratory diseases and causes of death following a natural pandemic event

**DOI:** 10.1371/journal.pgph.0006376

**Published:** 2026-07-15

**Authors:** Michael Sieber, Arne Traulsen

**Affiliations:** Department of Theoretical Biology, Max Planck Institute for Evolutionary Biology, Plön, Germany; City University of Hong Kong Jockey Club College of Veterinary Medicine and Life Sciences, HONG KONG

## Abstract

Seasonal respiratory infections typically surge within a limited time window, but what determines the exact timing within a given year is not well understood. Major causes of death show a similar seasonal pattern, but whether this pattern is linked to respiratory infections is unclear. The disruptions caused by the COVID-19 pandemic led to dramatic changes in the transmission dynamics of many respiratory pathogens, providing a unique opportunity to study the determinants of seasonal epidemics and the role of infectious diseases in driving seemingly unrelated causes of mortality. We analyzed publicly available data on weekly incidences of respiratory infections and all-cause mortality in Germany for the last 14 years. The analysis was complemented with results from an epidemiological model to determine the main drivers of seasonal timing. The timing of the seasonal epidemic of respiratory infections in Germany has been remarkably consistent for years, peaking during just few weeks in late winter. During the COVID-19 pandemic the seasonal surge in respiratory infections occurred substantially earlier, initially driven by the emerging COVID-19 and during later seasons by the resurgence of influenza and RSV. Remarkably, this shift was accompanied by a similar shift in the seasonality of all-cause mortality, and in particular mortality due to cardiovascular disease. The observed shift in epidemic timing is a consistent, but transient, outcome of disrupted epidemic seasonality, predictable from basic epidemiological principles. Our study reinforces that the buildup of susceptibles after the pandemic disruption was responsible for the shift in timing of the seasonal epidemic of respiratory infections. We show that more generally the rate of immune waning is the main determinant of the exact timing of seasonal epidemics of infectious diseases. The corresponding shift in seasonality of cardiovascular mortality suggests a connection between recent respiratory infections and severe cardiovascular events. This highlights the importance of monitoring individual infection history and improving vaccination coverage, in particular against influenza.

## Introduction

Many infectious respiratory diseases in human populations experience a seasonal variation in transmission, leading to recurring epidemics [1–5]. The non-pharmaceutical interventions (NPIs) implemented during the COVID-19 pandemic constituted a major disruption of the seasonality for many endemic respiratory diseases, resulting in wide-spread changes to their usual seasonal dynamics [6–11]. One of the most striking examples of this effect was the almost complete disappearance of influenza and respiratory syncytial virus (RSV) for one season or even longer [12–15].

It had been suggested during the COVID-19 pandemic that such disruptions can lead to changes in the severity and timing of the seasonal dynamics of the respective diseases [16–20]. Out-of-season waves of respiratory infections following COVID-19-associated NPIs have subsequently been reported from around the world [13,15,21–30], but these waves were not necessarily more severe than previous seasons [31].

Whether these unusual epidemic patterns are a predictable consequence of disruptions of seasonal infection dynamics and also translate to changes in temporal patterns of mortality has not been systematically evaluated. While the major drivers of disease seasonality such as changes in the abiotic environment, vector seasonality and seasonal changes in host behaviour [1,32–34] determine the overall seasonality of diseases, the determinants of the exact timing remain largely unknown [1].

With almost three full seasonal cycles having passed since the Public Health Emergency of International Concern due to COVID-19 has been declared over in May 2023, a more complete picture of the magnitude and duration of the disruptive changes of seasonal epidemics due to NPIs emerges. The interplay of an emerging respiratory disease and the disruption and resurgence of already circulating diseases provides a unique opportunity to study the drivers of seasonal forcing of respiratory diseases and the factors determining the seasonal timing of epidemics [1].

## Methods

### Data on respiratory infections

We analyzed publicly available data for weekly incidences (cases per 100.000 persons) of self-reported symptomatic respiratory infections [35], doctor’s visits for acute respiratory infections [36], and hospitalisations due to severe acute respiratory infections [37] in Germany for the last 10–12 years.

Additionally, we obtained weekly incidences of influenza, COVID-19, and RSV from the German database of cases of notifiable diseases using the SurvStat@RKI 2.0 application [38].

### Data on mortality

We also analyzed the timeseries of weekly all-cause mortality in Germany, publicly available from the German Federal Statistics Office [39,40]. The incidence of mortality per week (deaths per 100.000 persons) was calculated by dividing the absolute numbers of deaths by the total population of Germany obtained from the German Federal Statistics Office [41].

In addition, we obtained monthly data on the number of deaths attributed to specific causes from the German Federal Statistics Office. This data is available upon request from the Federal Statistics Office, and it is also included in the github repository accompanying this paper.

### SIR model

The transient shift in seasonal timing of respiratory diseases can be understood within an established SIR (susceptible-infectious-recovered) model with seasonal forcing. Such SIR or related SEIR (susceptible-exposed-infectious-recovered) models have been used to shed light on the epidemiological dynamics of seasonal diseases, including sudden transitions between different epidemiological patterns [42], and the effects of behavioural or environmental perturbations of the seasonal forcing [43,44].

In the simplest case, the deterministic SIR model describes the spread of an infectious disease in a population of constant size with the fraction of susceptible individuals *S*, the fraction of infected individuals *I*, and the fraction of recovered and immune individuals *R*, so that *S* + *I* + *R* = 1. Susceptible individuals get infected with the intrinsic transmission rate β(t) by infected individuals. The transmission rate varies with time, reflecting that seasonally changing environmental conditions influence the transmissibility of the disease. We assume a periodically driven transmission rate


$$\begin{array}{c} \beta (t)=\beta_{min}+(1-p)\;\left(\beta_{max}-\beta_{min}\right)\;\frac{1}{2}\;\left(1-\cos\left(\frac{2\pi }{52}t\right)\right)\;, \end{array}$$

which for *p* = 0 oscillates between a minimal transmission rate $\beta_{min}$ and a maximum transmission rate $\beta_{max}$. The period of the seasonal forcing is chosen as 52 weeks, with the maximum transmission rate occurring at *t* = 26 weeks, defined as the turn of the calendar year and mid-season.

The factor 1−p in this model reflects the reduction of the amplitude of the seasonal forcing due to non-pharmaceutical interventions (NPIs), so that *p* = 0 during a usual season in the absence of NPIs and 0<p≤1 during periods of NPIs. We assume that NPIs can not reduce transmission below the minimal rate $\beta_{min}$ even at 100% efficacy, but our results do not depend on the specific way NPI efficacy *p* affects transmission. Unless stated otherwise, NPIs reduce transmission by 30% (*p* = 0.3).

The ordinary differential equations (ODEs) describing this SIR model are


$$\begin{array}{cc} \text{susceptible}\;\frac{dS}{dt} & =-\beta (t)\;S\;I+\omega \;R\\ \text{infective}\;\frac{dI}{dt} & =\beta (t)\;S\;I-\rho \;I\\ \text{recovered}\;\frac{dR}{dt} & =\rho \;I-\omega \;R \end{array}$$ (1)

The goal of our model analysis is to understand the general factors influencing the timing of seasonal epidemics, rather than to predict the dynamics of any specific infectious disease. We thus fix the parameter values within the ranges that have previously been estimated for seasonal respiratory diseases. The minimal transmission rate is fixed at $\beta_{min}=0.6$, and the maximum transmission rate at $\beta_{max}=3$. After infection an individual remains infective for a duration of $\rho^{-1}=1$ week, after which it recovers and enters the fraction *R*. These values for transmission and recovery rates lie within the range estimated previously for seasonal influenza [32], RSV [45], and other respiratory viruses [46]. Waning immunity is the result of a complex interaction between the host’s immune system and antigenic variation of the causal pathogen, and the exact duration of protection is uncertain for many respiratory diseases [47]. Here we assume that recovered individuals are protected against re-infection for a duration of immunity of $\omega^{-1}=40$ weeks. This leads to annual epidemics and similar values have previously been used to describe the spread of seasonal influenza [43].

The deterministic ODE model (1) was numerically solved with Python using the *solve_ivp* function from the SciPy software library [48].

### Code and data availability

The code and all data used to generate the results and figures are available at https://github.com/misieber/arishift.

## Results

### Transient shift in timing of respiratory disease season

**Fig 1:**
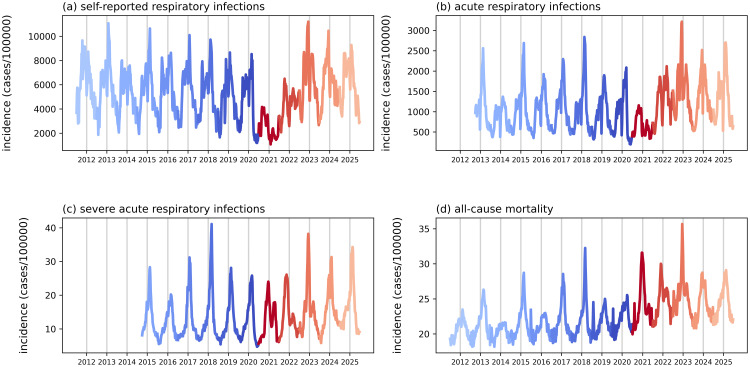
Timeseries data showing the strong seasonal component of respiratory infections and all-cause mortality in Germany. Pre-pandemic seasons are shown in blue and (post-)pandemic seasons in red colors. **(a)** Weekly incidence of self-reported, symptomatic respiratory infections (SRI). **(b)** Weekly incidence of acute respiratory infections (ARI). **(c)** Weekly incidence of hospitalized severe acute respiratory infections (SARI). **(d)** All-cause mortality as number of deaths per 100.000 per week. The winter peaks are often interspersed with a sharp summer peak, corresponding to heat-related mortality in particularly hot summers.

**Fig 2:**
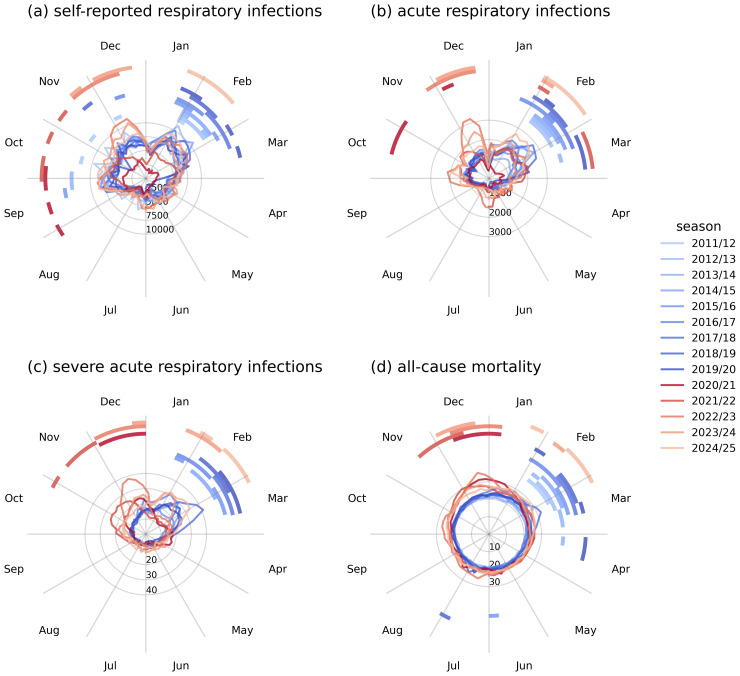
The same data as in Fig 1 in a polar plot to highlight the shift in the timing of seasonal epidemics. As in Fig 1, pre-pandemic seasons are shown in blue and (post-)pandemic seasons in red. The bars above the timeseries indicate the four weeks with the highest incidences in each season. Before the pandemic, those peak weeks occurred almost exclusively in February and March. During and after the pandemic they were shifted to December or even earlier. **(a)** Weekly incidence of self-reported, symptomatic respiratory infections (SRI). **(b)** Weekly incidence of acute respiratory infections (ARI). **(c)** Weekly incidence of hospitalized severe acute respiratory infections (SARI). **(d)** All-cause mortality as number of deaths per 100.000 per week.

The incidences of respiratory infections follow a clear seasonal pattern, increasing four to five-fold in late fall and winter compared to the summer months (Figs 1a-1c). This seasonal pattern was remarkably stable before the COVID-19 pandemic, with incidences consistently peaking during just a few weeks in February and March each year (Figs 2a-2c).

**Fig 3:**
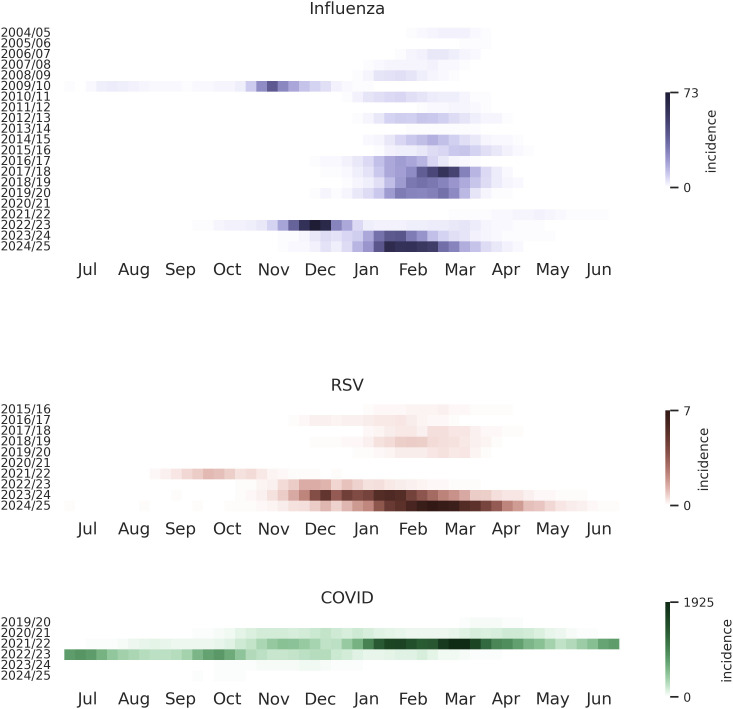
Weekly incidences of influenza, RSV and COVID-19 in Germany. Influenza and RSV very consistently peaked in February and March, except during the H1N1 influenza pandemic 2009/10 and the COVID-19 pandemic. For influenza and RSV we observe a gradual shift of the season back to its normal timing.

The NPIs implemented in 2020/21 during the first winter of the the COVID-19 pandemic in Germany resulted in a reduced transmission not only of Sars-CoV-2, but also of many other respiratory infections. For example, seasonal influenza and RSV were almost completely absent in the winter of 2020/21 (Fig 3). This reduced transmission of respiratory pathogens due to NPIs is reflected in a reduced number of self-reported respiratory infections and doctor’s visits for ARIs in the fall and winter of 2020/21 (Fig 1a, 1b). This reduction was found across all age groups, with weekly incidences reduced by 50% or more compared to previous seasons (S1–S3 Figs). The effect was even more striking for hospitalisations of 0–4 years olds, with no discernible seasonal increase of SARI cases at all in the winter of 2020/21 (S1 Fig).

Despite this anomalous season at the start of the COVID-19 pandemic, the overall epidemiological dynamics of ARI cases continued to be dominated by a clear seasonal signal (Fig 1a-1c), in particular for the severe cases that required hospitalisation (Fig 1c). At the same time, rapid evolution of SARS-CoV-2 led to out-of-season waves of emerging viral variants, most prominently the spread of the Alpha variant from January to April 2021, and the Omicron variant from January to March 2022. These waves appear as overlaying the dominating seasonal pattern (Fig 1a-1c).

While the overall seasonality of acute respiratory diseases appeared unchanged after the anomalous 2020/21 season, there was a striking shift in timing: In the first winter of the COVID-19 pandemic in 2020/21 and the two following seasons from 2021/22–2022/23, the onset and peak of respiratory disease season was shifted by several weeks, each occurring 8–12 weeks earlier than in all of the observed previous seasons (Figs 2a-2c).

For the 2020/21 winter, this shift was driven by the seasonally early epidemic spread of COVID-19 (Fig 3), which was responsible for the majority of ARI and SARI cases in the absence of influenza and RSV. After being absent in 2020/21, RSV re-emerged in September of 2021/22, very early in the season and almost simultaneously with the start of the next winter wave of COVID-19 (Fig 3). The COVID-19 pandemic was at this time mainly driven by the highly pathogenic Delta variant (S5 Fig).

As a consequence the incidence of hospitalized SARI cases already peaked in November, earlier than in any other observed season and 3–4 months before the usual pre-pandemic timing (Fig 2c). For the ARI cases the 2021/22 season is more complicated, as the highly transmissible, but less pathogenic Omicron variant began to rapidly replace the Delta variant in late 2021 and early 2022 (S5 Fig). This emerging variant lead to a peak in ARI cases in March 2022, which did not lead to a corresponding peak in hospitalisations, presumably due to the generally lower pathogenicity of the Omicron variant. In 2022/23 seasonal influenza, re-emerging after being almost completely absent in 2020/21 and 2021/22, had an early epidemic peak in December, 2–3 months before its usual peak in February or March (Fig 3). Simultaneously with influenza in 2022/23 the RSV epidemic occurred, two months later than the very early season in 2021/22 right after its re-emergence, but still two months earlier than usual (Fig 3).

This suggests a shift back towards the usual, pre-pandemic seasonal timing for RSV, a pattern that was confirmed for both RSV and influenza in the most recent season in 2023/24. A similar shift back to pre-pandemic seasonal patterns had also been reported in the US [28]. The 2023/24 season was characterized by a succession of COVID-19 early in the season from September to December, followed by seasonal influenza and RSV, which both peaked almost back at their normal timing in late January and early February (Fig 3). This pattern was confirmed in the 2024/25 season, suggesting that the shift in seasonal timing for previously circulating diseases in Germany was transient and was gradually reversed within one or two seasons.

### Corresponding shift in seasonality of mortality

The excess mortality associated with the COVID-19 pandemic has received a lot of attention, showing that the number of deaths was substantially higher than expected around the world [49]. But changes to the temporal dynamics of mortality have received relatively little systematic attention. In Germany, weekly mortality follows a clear seasonal pattern (Figs 1d, 2d), correlated with the weekly incidences of ARI and SARI cases. While peak mortality can vary by more than 30% season-by-season, the timing of the seasonal peak in mortality was remarkably stable before the COVID-19 pandemic. Mortality generally peaked in February or March, coinciding with the pre-pandemic seasonal peaks in ARI and SARI cases (Fig 2d). Naturally, the dynamics of all-cause mortality are dominated by the older age groups, and this clear seasonal pattern is absent from the youngest age groups (S1 Fig).

Strikingly, during and after the COVID-19 pandemic the seasonal peak in mortality occurred 2–3 months earlier, in December and early January (Fig 2d). This is a shift very similar to the one observed for respiratory infections and a strong signal of the effect the pandemic had not only on epidemiological processes, but also seasonal dynamics of mortality.

The observed shift in all-cause mortality is even more striking considering that deaths directly attributed to respiratory diseases account for no more than about 15% of the total annual mortality (S4 Fig). Intriguingly, mortality linked to cardiovascular disease, the main cause of death in Germany, also exhibits strong seasonal dynamics and usually peaks in late winter (S4 Fig). Seasonality of cardiovascular disease has been widely documented [50,51], and sometimes been attributed to direct effects of environmental factors [52,53]. The strong correlation with seasonal respiratory epidemics before the pandemic could also hint at a more direct link between the physiological effects of respiratory diseases and cardiovascular mortality [54–59]. But due to the strong correlation of these unrelated cardiovascular risk factors with a common environmental driver, a cause-and-effect relationship has been difficult to establish [54,58].

**Fig 4:**
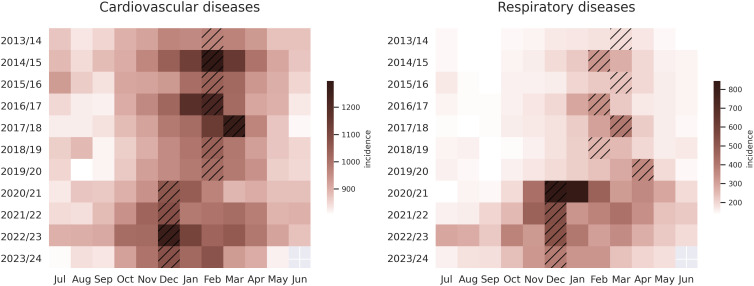
Monthly incidence of mortality due to respiratory diseases and cardiovascular diseases in Germany. The month with the highest annual mortality is highlighted. Mortality due to both causes consistently peaked in late winter up to the onset of the COVID-19 pandemic. During and after the pandemic mortality attributed to both causes shifted 2-3 months forward with the peak now occurring in December. This is in line with the earlier timing of the seasonal respiratory disease epidemic (Fig 2).

The substantial shift in the seasonal pattern of respiratory diseases provides an ideal opportunity to test if such a link exists. Looking at the monthly cardiovascular mortality for the last eleven years shows that up until the 2019/20 season, mortality consistently peaked in February or March, in line with respiratory infections (Fig 4). Now, if the seasonality of cardiovascular mortality is independently driven by environmental factors, we would expect the seasonal dynamics of heart attacks or strokes to remain unaffected by the disruptions caused by the COVID-19 pandemic. But what we actually observe during the pandemic seasons 2020/21–2022/23 is that the seasonal pattern of cardiovascular mortality shifted in lockstep with seasonal respiratory infections, with peak mortality now occurring in December (Fig 4). This corresponds to tens of thousands of deaths occurring 2–3 months earlier in the season than usual, signifying a major shift. For the most recent season the pattern shifted back towards its pre-pandemic timing (Fig 4), mirroring the gradual reverse shift of seasonal respiratory epidemics. This highly correlated seasonal shift of cardiovascular mortality and respiratory infections suggests that respiratory infections are one of the main drivers of seasonality in cardiovascular mortality.

### Shift in seasonal timing in an SIR model

**Fig 5:**
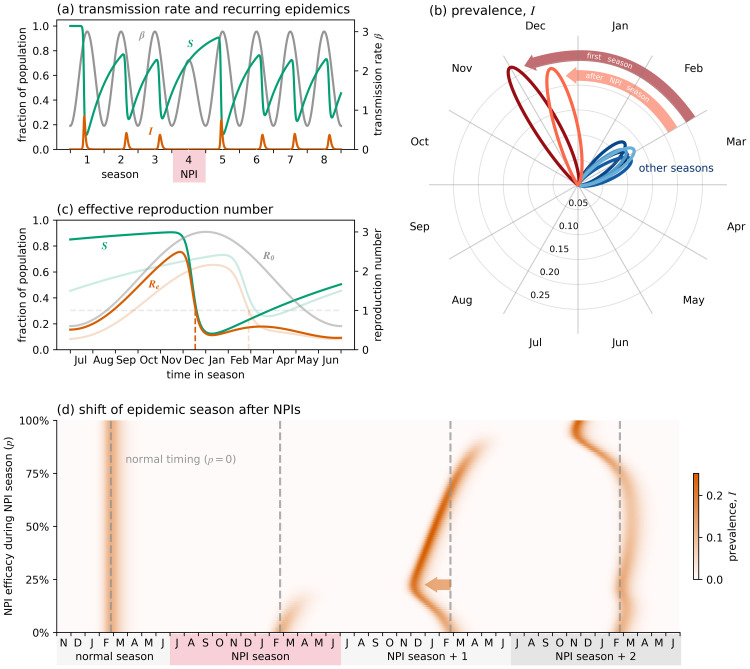
(a) Timeseries obtained from model (Eq 1). During the NPI season marked in red the maximum transmission rate $\beta_{max}$ is reduced by 30%. This results in the suppression of the seasonal epidemic and an earlier epidemic in the following season. (b) Same data as in (a) in a polar plot, highlighting the shift in peak timing. The seasonal epidemics after the initial introduction of the disease and right after the NPI season happen earlier than during other seasons. (c) The effective reproduction number $R_{e}$ (red lines) is higher earlier in the season after a prolonged buildup of susceptibles S (green lines) due to NPIs, which is similar to the scenario of a newly arising disease with limited pre-existing population-level immunity. The faded lines show the situation during a usual season without disruptions. The dashed red vertical lines mark the peak of the seasonal epidemic in the respective seasons, showing the shift forward after an NPI season. (d) Effect of different levels of NPI stringency on the shift of subsequent seasonal epidemics. The NPIs generally lead to earlier epidemics in the following season, unless NPIs are so effective that they almost eradicate the disease. In this case the following season can be slightly later and weaker than usual, or even be entirely skipped (here for p> >  ca. 75%). The disease resurgence two seasons later is then again earlier than usual.

A timeseries obtained from the numerical solution of model (1) is presented in Fig 5a, starting from an initially completely susceptible population. This corresponds to the scenario of a novel disease spreading in a population without pre-existing immunity, as for example at the onset of the COVID-19 pandemic. After introduction of the disease the seasonally varying transmission rate leads to seasonal epidemics with a very consistent timing (Fig 5b). For the chosen parameter values the peaks of the recurring epidemics are reached in a narrow time window just after the transmission rate has reached its seasonal maximum.

The very first epidemic is an exception to this pattern, as it starts much earlier in the seasonal cycle and reaches its maximum before maximum transmissibility has been reached at the turn of the year (Fig 5b). This can be understood in terms of the number of secondary infections caused by one infective individual, which in a completely susceptible population (S≈1) is determined by the basic reproduction number $R_{0}=\beta (t)/\rho$. This number is directly proportional to the intrinsic transmissibility β(t) of the disease, and thus it changes with the time of season. But this is only valid in a fully susceptible population and more generally the reproduction number not only depends on the transmission rate β(t), but also on the current size of the fraction *S* of the population that is susceptible. This leads to the effective reproduction number $R_{e}=R_{0}\;S$, where both the basic reproduction number *R*_0_ and the susceptible fraction *S* change dynamically in time.

The disease will only spread and cause an epidemic if $R_{e}>1$, which in the model (Eq 1) exactly corresponds to a positive growth rate of the infective fraction *I*. In this case, the size of the susceptible fraction *S* decreases as more individuals become infected and eventually recover, until $R_{e}$ falls below 1 again. At this point the epidemic has reached its seasonal peak and the infective fraction starts to decrease. After some time, the susceptible fraction will start to increase again due to waning immunity until the cycle begins again.

Since outside of NPI-periods the transmission rate β has fixed temporal dynamics, it is the dynamic size of the susceptible fraction *S* that determines the exact timing of when the effective reproduction number will be above or below 1. Generally, the larger the fraction *S* is earlier in the seasonal cycle, the earlier the effective reproduction number will cross this threshold and the earlier the epidemic will start relative to its underlying seasonal forcing. After introduction of a disease into a fully susceptible population (S≈1) the effective reproduction number $R_{e}$ is close to the basic reproduction number $R_{0}=\beta (t)/\rho$, and consequently the epidemic starts at the earliest possible time, i.e., as soon as β(t)/ρ>1 (Fig 5c).

In the subsequent seasons, when there is some amount of pre-existing immunity on the population level (*S* < 1), the effective reproduction number $R_{e}<R_{0}$ governs the infection dynamics and thus the epidemic will start and peak later in the seasonal cycle (Fig 5c). Due to the constant rate of immune waning ω and in the absence of other disruptions, the increase of susceptibles between two consecutive epidemic peaks follows a very regular pattern. This results in subsequent epidemics having a very consistent timing later in the seasonal cycle compared to the initial season (Fig 5b).

If an epidemic season is reduced or entirely skipped due to NPIs, this consistent temporal pattern is disrupted. After an NPI period and a skipped seasonal epidemic, the buildup of susceptibles in the population due to prolonged immune waning leads to a higher $R_{e}$ earlier in the season (Fig 5c). This in turn results in an earlier onset of the corresponding seasonal epidemic, which will generally fall somewhere between the timing of the very first epidemic season and the usual, later timing of unperturbed seasons (Fig 5b).

The extent of the shift away from the usual seasonal timing depends on the size of the susceptible fraction reached during and after the NPI period. This buildup of susceptibles ultimately depends on the ability of the NPIs to suppress the disease, in the model described by the efficacy *p* of NPIs. Varying the efficacy *p* of the NPIs shows that the seasonal epidemic is effectively contained if the transmission rate is reduced by 20% or more (Fig 5d). At the same time the following season is shifted forward towards an earlier timing. For the chosen parametrization this shift is most pronounced for an NPI efficacy of ca. 25%, but the forward-shift is observed for the larger part of the efficacy range up to p≈70% (Fig 5d). Above p≈70%, the seasonal epidemic is slightly delayed, despite the effective reproduction number $R_{e}$ becoming larger than one earlier in the season.

This delay arises because with very effective NPIs the prevalence *I* drops so low that the exponential growth rate of the disease is initially so low that a noticeable epidemic develops only later in the season. If the NPIs are so effective that the disease is almost driven to extinction (*p* > 90%), the exponential growth rate is in fact so low that there is not enough time for the infection to grow into an epidemic within the seasonal window. In this case the epidemic in the first season after the NPIs is skipped entirely and the disease re-emerges in the second season after the NPIs. This resurgence then again happens much earlier compared to the usual epidemic timing (Fig 5d). This delay or skipping of an additional season after very effective NPIs can be reduced or even reversed if there is a small, constant influx of infecteds, which prevents the prevalence *I* from dropping to extremely low values.

The exact size and timing of seasonal disease outbreaks also depends on the dynamics of the underlying seasonal forcing. But the general pattern of diseases spreading earlier in their seasonal cycles either directly after introduction or after reduced transmission in a previous season is generally robust to variations in the minimum and maximum transmission rates (S6 Fig). If, however, maximum transmission rates are very high the usual seasonal peaks already happen relatively early, and disruptions by NPIs have no potential to move them much further forward (S6 Fig).

Another result from this simple model is that if waning of immunity happens on a much faster time scale compared to the seasonal cycle ($\omega^{-1}\ll 52$ weeks), a similar shift of the epidemic timing following a perturbation of the seasonal forcing would not be observed (S7 Fig). In this case, following an epidemic the population would quickly be made up mostly of susceptibles again, and the next season would thus again start at the earliest possible time, solely determined by the intrinsic transmission rate β(t).

## Discussion

In general, two factors determine if and when seasonal epidemics occur: (i) seasonal variation of pathogen transmission driven mainly by environmental factors and (ii) the fraction of the population that is susceptible [1]. But the relative importance of these factors and how they work together to determine the exact timing is not clear. The NPIs associated with the COVID-19 pandemic and the resulting disruption of the pre-pandemic seasonal pattern has allowed us to disentangle these factors in more detail.

We observed a significant shift in the timing of seasonal respiratory disease epidemics in Germany during the fall and winter of 2021/22 and 2022/23. A corresponding shift is also observed in the seasonal pattern of all-cause mortality. Together with the complete absence of the most important respiratory diseases during the 2020/21 and 2012/22 seasons, this shift is one of the clearest signals of the SARS-CoV-2 pandemic and associated mitigation measures.

This shift is even more profound considering that the timing of seasonal epidemics in Germany has been remarkably consistent, with highest incidences typically occurring within a relatively narrow time window in February and March. In fact, in the last 20 years there is only one precedent of a similar seasonal shift in Germany, caused by the novel 2009 H1N1 influenza which lead to a global pandemic [60]. This early-onset influenza epidemic also led to a transient change in the timing of seasonal epidemics of RSV and other respiratory diseases. In this case the change in timing was not as pronounced and not necessarily all in the same direction [60], which may be more a result of direct viral interference rather than the more limited and heterogenous implementation of NPIs during this pandemic [61].

We supplemented our analysis of respiratory disease epidemics in Germany with results from a seasonally-forced SIR model. This shows that seasonal variation of transmission creates the necessary window of opportunity for epidemics, but it is the availability of susceptibles at the onset of the season that determines the exact timing of the epidemic within this window. The susceptible fraction of the population increases between consecutive epidemics as a result of a gradual reduction of antibody levels and antigenic shift of the pathogen [47]. For an annually occurring epidemic, skipping of one season then leads to a larger fraction of susceptibles at the start of the next epidemic window. An analysis of the effective reproduction number within the context of an SIR model (Eq 1) then predicts an earlier onset and peak of the epidemic (Fig 5a), matching the epidemiological data (Fig 1). This view on the temporal dynamics of the susceptible pool and the effective reproduction number is analogous to the notions of overcompensation and return time recently introduced to analyze the conditions for mutual invasibility and co-circulation of pathogens [62]. Our results are also related to the analysis of a similar SIR model which linked the availability of susceptibles after a seasonal epidemic to whether another epidemic would be observed in the following season [63].

It is important to note that this shift in timing is not the result of an individual immunity debt or otherwise weaker immune system, since individual differences due to previous exposures to the pathogen is not part of the model. Instead, any changes to the usual seasonal dynamics are the result of a loss of population-level immunity and a greater pool of susceptible hosts, not because of individually more severe infections.

The SIR model also predicts that this increased pool of available hosts is depleted quickly during just one season, bringing the size of the susceptible fraction back to its usual pre-NPI level two seasons after the skipped season (Fig 5a). The model thus predicts a relatively quick shift back to the normal timing and severity for seasonal respiratory epidemics, which is indeed the case for influenza and RSV in Germany (Fig 3).

Since a greater fraction of susceptibles leads to a higher effective reproduction number earlier in the season, the pathogen starts to spread earlier in its seasonal window and the predicted shift in epidemic timing is generally towards earlier in the season. But if the pathogen has been driven almost to extinction during the NPI season, even early-onset growth may not be enough to lead to a noticeable increase in disease incidence during the next season. In this case, repeated cryptic spread over one or more seasons is necessary before a noticeable epidemic occurs again. This form of delayed resurgence has for example been described for bacterial respiratory infections with *Mycoplasma pneumoniae* [64].

A shift of 2–3 months in the seasonal timing of major respiratory diseases has profound practical implications. Doctors and hospitals may see a surge of cases when they do not usually expect them, leading to staffing and resource shortages. This is exacerbated by the shifted peaking of acute and severe respiratory cases during December in Germany, overlapping with the seasonal holidays. It also highlights the need to anticipate an earlier start to seasonal vaccination campaigns, such as against influenza, if the preceding season was unusually mild or entirely skipped. Epidemiologists and public health practitioners need to be aware that comparing epidemiological data from the current season to the same time point in previous seasons is not meaningful if a major disruption of the usual seasonal pattern has occurred.

Beyond the direct impact of the COVID-19 pandemic on seasonal respiratory infections one of the most striking observations is the corresponding change in the seasonal dynamics of mortality (Fig 2d). The shift mirrors the change in respiratory disease seasonality, and is mainly driven by a substantial shift in the seasonality of mortality due to cardiovascular disease (Fig 4). This supports the hypothesis that viral respiratory infections are a major risk factor for cardiovascular disease, mediated by the effects of viral infections and associated inflammatory responses [65]. Future research combining individual infection histories and cardiovascular event records would help to further confirm this association, and to rule out the influence of other pandemic-related factors, such as changes in medical service accessibility and individual behaviour. Our results also further highlight the importance of timely vaccinations against seasonal respiratory infections as a public health measure beyond preventing acute viral infections [66,67].

More than two full epidemic seasons have passed since the WHO declared the global COVID-19 pandemic over in May 2023. As we have seen from the data on the incidences of respiratory infections, the overall seasonal dynamics of respiratory infections is returning back to its pre-pandemic pattern. Seasonal influenza and RSV in particular have returned to surging in from late January to March during the 2023/24 and 2024/25 seasons (Fig 3).

Seasonal forcing of transmission also plays a role for SARS-CoV-2 [68], but here the situation remains more complicated due to newly arising variants, rapidly waning immunity and very high effective reproduction number. Despite almost the entire population of Germany having been in contact with either SARS-CoV-2 or a vaccine by mid-2022 [69], the inherently much larger transmissibility of SARS-CoV-2 makes it prone to spread earlier in the season than other respiratory diseases. In the 2023/24 and 2024/25 the seasonal SARS-CoV-2 epidemic was starting from as early as late August and was running out by the end of December (Fig 3). For the time being we should thus expect a succession of early seasonal onset of COVID-19 in late summer and fall, followed by seasonal influenza and RSV closer to their usual time windows of late winter and early spring. This suggests that a combined vaccination campaign against influenza and COVID-19 in late fall may actually be too late for COVID-19, and vaccination against COVID-19 should ideally be rolled out already in late summer.

While we have focused on the epidemiology in Germany, repeatable and consistent timing is a hallmark of seasonal respiratory disease epidemics in many temperate regions [70]. At the same time there is evidence for shifts in epidemic timing worldwide, suggesting that our observations hold more generally. In the United States, for example, the flu season 2022 started earlier than usual [71,72], before returning to its usual timing a season later [73].

The minimal SIR model neglects evolutionary aspects of seasonal epidemics arising from the intertwined processes of waning immunity on the host side and antigenic variation on the pathogen side [74,75]. But the very repeatable timing of seasonal epidemics suggests that such processes do not generally lead to large-scale shifts in the timing and peak of epidemics. This either requires the emergence of a novel pathogen into largely immune-naive population or a major disruption of the usual seasonal dynamics, for example through widespread implementation of sufficiently strict NPIs.

Our analysis of epidemiological data and an SIR model highlights that disruptions to the usual seasonal dynamics of respiratory infections not only affect the severity, but even more so the otherwise very consistent timing of seasonal epidemics. A basic SIR model reproduces this shift across a wide range of scenarios, showing that such large scale patterns can be explained by the population level epidemiology of shifting susceptible and infective sub-populations. Beyond explaining the seasonal shift following the SARS-CoV-2 pandemic our analysis shows more generally that, within the exogenously determined window of epidemic potential, endogenous variables such as the availability of susceptibles are key for the exact timing of epidemics.

## Supporting information

S1 Fig Weekly incidences of respiratory infections in Germany, age group 0–4. (PDF)

S2 Fig Weekly incidences of respiratory infections in Germany, age group 35–59. (PDF)

S3 Fig Weekly incidences of respiratory infections in Germany, age group >60. (PDF)

S4 Fig Timeseries of monthly incidences of major causes of death in Germany. (PDF)

S5 Fig Timeline of major SARS-CoV-2 variants in Germany. (PDF)

S6 Fig Additional modelling results for varying transmission rates. (PDF)

S7 Fig Additional modelling results for shorter duration of immunity. (PDF)

## References

[1] Martinez ME. The calendar of epidemics: Seasonal cycles of infectious diseases. PLoS Pathog. 2018;14(11):e1007327. doi: 10.1371/journal.ppat.1007327 30408114 PMC6224126

[2] Li Y, Reeves RM, Wang X, Bassat Q, Brooks WA, Cohen C, et al. Global patterns in monthly activity of influenza virus, respiratory syncytial virus, parainfluenza virus, and metapneumovirus: a systematic analysis. Lancet Glob Health. 2019;7(8):e1031–45. doi: 10.1016/S2214-109X(19)30264-5 31303294

[3] Moriyama M, Hugentobler WJ, Iwasaki A. Seasonality of Respiratory Viral Infections. Annu Rev Virol. 2020;7(1):83–101. doi: 10.1146/annurev-virology-012420-022445 32196426

[4] Neumann G, Kawaoka Y. Seasonality of influenza and other respiratory viruses. EMBO Mol Med. 2022;14(4):e15352. doi: 10.15252/emmm.202115352 35157360 PMC8988196

[5] García-Arroyo L, Prim N, Del Cuerpo M, Marín P, Roig MC, Esteban M, et al. Prevalence and seasonality of viral respiratory infections in a temperate climate region: A 24-year study (1997–2020). Influenza and Other Respiratory Viruses. 2022;16(4):756–66. 35170253 10.1111/irv.12972 PMC9178050

[6] Gomez GB, Mahé C, Chaves SS. Uncertain effects of the pandemic on respiratory viruses. Science. 2021;372(6546):1043–4. doi: 10.1126/science.abh3986 34083477

[7] Tang JW, Bialasiewicz S, Dwyer DE, Dilcher M, Tellier R, Taylor J, et al. Where have all the viruses gone? Disappearance of seasonal respiratory viruses during the COVID-19 pandemic. J Med Virol. 2021;93(7):4099–101. doi: 10.1002/jmv.26964 33760278 PMC8250511

[8] Redlberger-Fritz M, Kundi M, Aberle SW, Puchhammer-Stöckl E. Significant impact of nationwide SARS-CoV-2 lockdown measures on the circulation of other respiratory virus infections in Austria. J Clin Virol. 2021;137:104795. doi: 10.1016/j.jcv.2021.104795 33761423 PMC7962988

[9] Rodgers L, Sheppard M, Smith A, Dietz S, Jayanthi P, Yuan Y, et al. Changes in Seasonal Respiratory Illnesses in the United States During the Coronavirus Disease 2019 (COVID-19) Pandemic. Clin Infect Dis. 2021;73(Suppl 1):S110–7. doi: 10.1093/cid/ciab311 33912902 PMC8135472

[10] Ullrich A, Schranz M, Rexroth U, Hamouda O, Schaade L, Diercke M, et al. Impact of the COVID-19 pandemic and associated non-pharmaceutical interventions on other notifiable infectious diseases in Germany: An analysis of national surveillance data during week 1-2016 - week 32-2020. Lancet Reg Health Eur. 2021;6:100103. doi: 10.1016/j.lanepe.2021.100103 34557831 PMC8454829

[11] Hsu H-T, Huang F-L, Ting P-J, Chang C-C, Chen P-Y. The epidemiological features of pediatric viral respiratory infection during the COVID-19 pandemic in Taiwan. J Microbiol Immunol Infect. 2022;55(6 Pt 1):1101–7. doi: 10.1016/j.jmii.2021.09.017 34756671 PMC8501510

[12] Feng L, Zhang T, Wang Q, Xie Y, Peng Z, Zheng J, et al. Impact of COVID-19 outbreaks and interventions on influenza in China and the United States. Nat Commun. 2021;12(1):3249. doi: 10.1038/s41467-021-23440-1 34059675 PMC8167168

[13] Kuitunen I, Artama M, Haapanen M, Renko M. Respiratory virus circulation in children after relaxation of COVID-19 restrictions in fall 2021-A nationwide register study in Finland. J Med Virol. 2022;94(9):4528–32. doi: 10.1002/jmv.27857 35577532 PMC9347728

[14] Dhanasekaran V, Sullivan S, Edwards KM, Xie R, Khvorov A, Valkenburg SA, et al. Human seasonal influenza under COVID-19 and the potential consequences of influenza lineage elimination. Nat Commun. 2022;13(1):1721. doi: 10.1038/s41467-022-29402-5 35361789 PMC8971476

[15] Stein RT, Zar HJ. RSV through the COVID-19 pandemic: Burden, shifting epidemiology, and implications for the future. Pediatr Pulmonol. 2023;58(6):1631–9. doi: 10.1002/ppul.26370 36811330

[16] Baker RE, Park SW, Yang W, Vecchi GA, Metcalf CJE, Grenfell BT. The impact of COVID-19 nonpharmaceutical interventions on the future dynamics of endemic infections. Proc Natl Acad Sci U S A. 2020;117(48):30547–53. doi: 10.1073/pnas.2013182117 33168723 PMC7720203

[17] Zheng Z, Pitzer VE, Shapiro ED, Bont LJ, Weinberger DM. Estimation of the Timing and Intensity of Reemergence of Respiratory Syncytial Virus Following the COVID-19 Pandemic in the US. JAMA Netw Open. 2021;4(12):e2141779. doi: 10.1001/jamanetworkopen.2021.41779 34913973 PMC8678706

[18] Koltai M, Krauer F, Hodgson D, van Leeuwen E, Treskova-Schwarzbach M, Jit M, et al. Determinants of RSV epidemiology following suppression through pandemic contact restrictions. Epidemics. 2022;40:100614. doi: 10.1016/j.epidem.2022.100614 35901639 PMC9301974

[19] Messacar K, Baker RE, Park SW, Nguyen-Tran H, Cataldi JR, Grenfell B. Preparing for uncertainty: endemic paediatric viral illnesses after COVID-19 pandemic disruption. Lancet. 2022;400(10364):1663–5. doi: 10.1016/S0140-6736(22)01277-6 35843260 PMC9282759

[20] Cheng W, Zhou H, Ye Y, Chen Y, Jing F, Cao Z, et al. Future trajectory of respiratory infections following the COVID-19 pandemic in Hong Kong. Chaos. 2023;33(1):013124. doi: 10.1063/5.0123870 36725657

[21] Martinez PP, Li J, Cortes CP, Baker RE, Mahmud AS. The Return of Wintertime Respiratory Virus Outbreaks and Shifts in the Age Structure of Incidence in the Southern Hemisphere. Open Forum Infect Dis. 2022;9(12):ofac650. doi: 10.1093/ofid/ofac650 36519120 PMC9745764

[22] Chow EJ, Uyeki TM, Chu HY. The effects of the COVID-19 pandemic on community respiratory virus activity. Nat Rev Microbiol. 2023;21(3):195–210. doi: 10.1038/s41579-022-00807-9 36253478 PMC9574826

[23] Buchholz U, Lehfeld A-S, Tolksdorf K, Cai W, Reiche J, Biere B, et al. Respiratory infections in children and adolescents in Germany during the COVID-19 pandemic. J Health Monit. 2023;8(2):20–38. doi: 10.25646/11437 37408711 PMC10318561

[24] Alzaydi M, Alosaimi A, Alghamdi AA, Bamogaddam IY, Altassan MA, Almazrua A, et al. Changes in seasonal respiratory viral infections among pediatric population around the COVID-19 pandemic; 2019-2023. Eur J Clin Microbiol Infect Dis. 2024;43(8):1589–96. doi: 10.1007/s10096-024-04860-5 38814498

[25] Maison N, Omony J, Rinderknecht S, Kolberg L, Meyer-Bühn M, von Mutius E, et al. Old foes following news ways?-Pandemic-related changes in the epidemiology of viral respiratory tract infections. Infection. 2024;52(1):209–18. doi: 10.1007/s15010-023-02085-w 37644253 PMC10811157

[26] Eden J-S, Sikazwe C, Xie R, Deng Y-M, Sullivan SG, Michie A, et al. Off-season RSV epidemics in Australia after easing of COVID-19 restrictions. Nat Commun. 2022;13(1):2884. doi: 10.1038/s41467-022-30485-3 35610217 PMC9130497

[27] Disease Prevention EC, Control. Intensified circulation of respiratory syncytial virus (RSV) and associated hospital burden in the EU/EEA. Stockholm: ECDC. 2022.

[28] Hamid S, Winn A, Parikh R, Jones JM, McMorrow M, Prill MM, et al. Seasonality of Respiratory Syncytial Virus - United States, 2017-2023. MMWR Morb Mortal Wkly Rep. 2023;72(14):355–61. doi: 10.15585/mmwr.mm7214a1 37022977 PMC10078848

[29] Löwensteyn YN, Zheng Z, Rave N, Bannier MAGE, Billard M-N, Casalegno J-S, et al. Year-Round Respiratory Syncytial Virus Transmission in The Netherlands Following the COVID-19 Pandemic: A Prospective Nationwide Observational and Modeling Study. J Infect Dis. 2023;228(10):1394–9. doi: 10.1093/infdis/jiad282 37477906 PMC10640768

[30] Rios-Guzman E, Simons LM, Dean TJ, Agnes F, Pawlowski A, Alisoltanidehkordi A, et al. Deviations in RSV epidemiological patterns and population structures in the United States following the COVID-19 pandemic. Nat Commun. 2024;15(1):3374. doi: 10.1038/s41467-024-47757-9 38643200 PMC11032338

[31] Saravanos GL, Hu N, Homaira N, Muscatello DJ, Jaffe A, Bartlett AW, et al. RSV Epidemiology in Australia Before and During COVID-19. Pediatrics. 2022;149(2):e2021053537. doi: 10.1542/peds.2021-053537 35083489

[32] Shaman J, Pitzer VE, Viboud C, Grenfell BT, Lipsitch M. Absolute humidity and the seasonal onset of influenza in the continental United States. PLoS Biol. 2010;8(2):e1000316. doi: 10.1371/journal.pbio.1000316 20186267 PMC2826374

[33] Price RHM, Graham C, Ramalingam S. Association between viral seasonality and meteorological factors. Sci Rep. 2019;9(1):929. doi: 10.1038/s41598-018-37481-y 30700747 PMC6353886

[34] Liang Y, Sun Z, Hua W, Li D, Han L, Liu J, et al. Spatiotemporal effects of meteorological conditions on global influenza peaks. Environ Res. 2023;231(Pt 2):116171. doi: 10.1016/j.envres.2023.116171 37230217

[35] Buchholz U, Lehfeld A, Loenenbach A, Prahm K, Preuß U, Stepanovich-Falke A. GrippeWeb - Daten des Wochenberichts. Zenodo. 2026. 10.5281/zenodo.18977552

[36] Goerlitz L, Tolksdorf K, Prahm K, Preuß U, Krupka S, Buchholz U. ARE-Konsultationsinzidenz. Zenodo. 2026. 10.5281/zenodo.12178537

[37] Tolksdorf K, Goerlitz L, Gvaladze T, Haas W, Buda S. Sari-hospitalisierungsinzidenz. Zenodo. 2026. 10.5281/zenodo.12178860

[38] Robert Koch Institute. SurvStat@RKI 2.0, deadline: 14.10.2025. Robert Koch Institute. 2026. https://survstat.rki.de

[39] Statistischer Bericht: Sterbefälle nach Tagen, Wochen und Monaten - endgültige Daten 2000-2020. Statistisches Bundesamt. 2025. https://www.destatis.de/DE/Themen/Gesellschaft-Umwelt/Bevoelkerung/Sterbefaelle-Lebenserwartung/Publikationen/Downloads-Sterbefaelle/statistischer-bericht-sterbefaelle-tage-wochen-monate-endg-5126108.html

[40] Statistischer Bericht: Sterbefälle nach Tagen, Wochen und Monaten - 2021-2026. Statistisches Bundesamt. 2026. https://www.destatis.de/DE/Themen/Gesellschaft-Umwelt/Bevoelkerung/Sterbefaelle-Lebenserwartung/Publikationen/Downloads-Sterbefaelle/statistischer-bericht-sterbefaelle-tage-wochen-monate-aktuell-5126109.html

[41] Statistisches B. Population: Germany, reference date, age. 2026. https://www-genesis.destatis.de/datenbank/online/statistic/12411/table/12411-0005

[42] Earn DJ, Rohani P, Bolker BM, Grenfell BT. A simple model for complex dynamical transitions in epidemics. Science. 2000;287(5453):667–70. doi: 10.1126/science.287.5453.667 10650003

[43] Baker RE, Saad-Roy CM, Park SW, Farrar J, Metcalf CJE, Grenfell BT. Long-term benefits of nonpharmaceutical interventions for endemic infections are shaped by respiratory pathogen dynamics. Proc Natl Acad Sci U S A. 2022;119(49):e2208895119. doi: 10.1073/pnas.2208895119 36445971 PMC9894244

[44] Ruan W, Liang Y, Sun Z, An X. Climate warming and influenza dynamics: the modulating effects of seasonal temperature increases on epidemic patterns. npj Clim Atmos Sci. 2025;8(1). doi: 10.1038/s41612-025-00968-3

[45] Weber A, Weber M, Milligan P. Modeling epidemics caused by respiratory syncytial virus (RSV). Math Biosci. 2001;172(2):95–113. doi: 10.1016/s0025-5564(01)00066-9 11520501

[46] Hawkes MT, Lee BE, Kanji JN, Zelyas N, Wong K, Barton M. Seasonality of respiratory viruses at northern latitudes. JAMA Network Open. 2021;4(9):e2124650. doi: 10.1001/jamanetworkopen.2021.24650 PMC8446819 34529066

[47] Siggins MK, Thwaites RS, Openshaw PJM. Durability of Immunity to SARS-CoV-2 and Other Respiratory Viruses. Trends Microbiol. 2021;29(7):648–62. doi: 10.1016/j.tim.2021.03.016 33896688 PMC8026254

[48] Virtanen P, Gommers R, Oliphant TE, Haberland M, Reddy T, Cournapeau D, et al. SciPy 1.0: fundamental algorithms for scientific computing in Python. Nat Methods. 2020;17(3):261–72. doi: 10.1038/s41592-019-0686-2 32015543 PMC7056644

[49] Msemburi W, Karlinsky A, Knutson V, Aleshin-Guendel S, Chatterji S, Wakefield J. The WHO estimates of excess mortality associated with the COVID-19 pandemic. Nature. 2023;613(7942):130–7. doi: 10.1038/s41586-022-05522-2 36517599 PMC9812776

[50] Marti-Soler H, Gonseth S, Gubelmann C, Stringhini S, Bovet P, Chen P-C, et al. Seasonal variation of overall and cardiovascular mortality: a study in 19 countries from different geographic locations. PLoS One. 2014;9(11):e113500. doi: 10.1371/journal.pone.0113500 25419711 PMC4242652

[51] Stewart S, Keates AK, Redfern A, McMurray JJV. Seasonal variations in cardiovascular disease. Nat Rev Cardiol. 2017;14(11):654–64. doi: 10.1038/nrcardio.2017.76 28518176

[52] Marti-Soler H, Gubelmann C, Aeschbacher S, Alves L, Bobak M, Bongard V, et al. Seasonality of cardiovascular risk factors: an analysis including over 230 000 participants in 15 countries. Heart. 2014;100(19):1517–23. doi: 10.1136/heartjnl-2014-305623 24879630

[53] Wyse CA, Celis Morales CA, Ward J, Lyall D, Smith DJ, Mackay D, et al. Population-level seasonality in cardiovascular mortality, blood pressure, BMI and inflammatory cells in UK biobank. Ann Med. 2018;50(5):410–9. doi: 10.1080/07853890.2018.1472389 29724143

[54] Madjid M, Naghavi M, Litovsky S, Casscells SW. Influenza and cardiovascular disease: a new opportunity for prevention and the need for further studies. Circulation. 2003;108(22):2730–6. doi: 10.1161/01.CIR.0000102380.47012.92 14610013

[55] Madjid M, Miller CC, Zarubaev VV, Marinich IG, Kiselev OI, Lobzin YV, et al. Influenza epidemics and acute respiratory disease activity are associated with a surge in autopsy-confirmed coronary heart disease death: results from 8 years of autopsies in 34,892 subjects. Eur Heart J. 2007;28(10):1205–10. doi: 10.1093/eurheartj/ehm035 17440221 PMC7108465

[56] Mamas MA, Fraser D, Neyses L. Cardiovascular manifestations associated with influenza virus infection. Int J Cardiol. 2008;130(3):304–9. doi: 10.1016/j.ijcard.2008.04.044 18625525

[57] Kwok CS, Aslam S, Kontopantelis E, Myint PK, Zaman MJS, Buchan I, et al. Influenza, influenza-like symptoms and their association with cardiovascular risks: a systematic review and meta-analysis of observational studies. Int J Clin Pract. 2015;69(9):928–37. doi: 10.1111/ijcp.12646 25940136 PMC7165588

[58] Nguyen JL, Yang W, Ito K, Matte TD, Shaman J, Kinney PL. Seasonal Influenza Infections and Cardiovascular Disease Mortality. JAMA Cardiol. 2016;1(3):274–81. doi: 10.1001/jamacardio.2016.0433 27438105 PMC5158013

[59] Kytömaa S, Hegde S, Claggett B, Udell JA, Rosamond W, Temte J, et al. Association of Influenza-like Illness Activity With Hospitalizations for Heart Failure: The Atherosclerosis Risk in Communities Study. JAMA Cardiol. 2019;4(4):363–9. doi: 10.1001/jamacardio.2019.0549 30916717 PMC6484790

[60] van Asten L, Bijkerk P, Fanoy E, van Ginkel A, Suijkerbuijk A, van der Hoek W, et al. Early occurrence of influenza A epidemics coincided with changes in occurrence of other respiratory virus infections. Influenza Other Respir Viruses. 2016;10(1):14–26. doi: 10.1111/irv.12348 26369646 PMC4687500

[61] Li K, Thindwa D, Weinberger DM, Pitzer VE. The role of viral interference in shaping RSV epidemics following the 2009 H1N1 influenza pandemic. 2024. 10.1111/irv.70111 PMC12022500 40275825

[62] Park SW, Cobey S, Metcalf CJE, Levine JM, Grenfell BT. Predicting pathogen mutual invasibility and co-circulation. Science. 2024;386(6718):175–9. doi: 10.1126/science.adq0072 39388572

[63] Stone L, Olinky R, Huppert A. Seasonal dynamics of recurrent epidemics. Nature. 2007;446(7135):533–6. doi: 10.1038/nature05638 17392785

[64] Park SW, Noble B, Howerton E, Nielsen BF, Jiudice SS, Ambroggio L. Predicting the impact of non-pharmaceutical interventions against COVID-19 on Mycoplasma pneumoniae in the United States. medRxiv. 2024;2024:2024–08. 10.1016/j.epidem.2024.100808 39642758

[65] Kawai K, Muhere CF, Lemos EV, Francis JM. Viral Infections and Risk of Cardiovascular Disease: Systematic Review and Meta-Analysis. J Am Heart Assoc. 2025;14(21):e042670. doi: 10.1161/JAHA.125.042670 41160032 PMC12684801

[66] Maniar YM, Al-Abdouh A, Michos ED. Influenza Vaccination for Cardiovascular Prevention: Further Insights from the IAMI Trial and an Updated Meta-analysis. Curr Cardiol Rep. 2022;24(10):1327–35. doi: 10.1007/s11886-022-01748-8 35876953 PMC9310360

[67] Heidecker B, Libby P, Vassiliou VS, Roubille F, Vardeny O, Hassager C, et al. Vaccination as a new form of cardiovascular prevention: a European Society of Cardiology clinical consensus statement: with the contribution of the European Association of Preventive Cardiology (EAPC), the Association for Acute CardioVascular Care (ACVC), and the Heart Failure Association (HFA) of the ESC. European Heart Journal. 2025. doi: ehaf384 10.1093/eurheartj/ehaf384 40582710

[68] Neher RA, Dyrdak R, Druelle V, Hodcroft EB, Albert J. Potential impact of seasonal forcing on a SARS-CoV-2 pandemic. Swiss Med Wkly. 2020;150:w20224. doi: 10.4414/smw.2020.20224 32176808

[69] Maier BF, Rose AH, Burdinski A, Klamser P, Neuhauser H, Wichmann O, et al. Estimating the share of SARS-CoV-2-immunologically naïve individuals in Germany up to June 2022. Epidemiol Infect. 2023;151:e38. doi: 10.1017/S0950268823000195 36789785 PMC10028997

[70] Servadio JL, Thai PQ, Choisy M, Boni MF. Repeatability and timing of tropical influenza epidemics. PLoS Comput Biol. 2023;19(7):e1011317. doi: 10.1371/journal.pcbi.1011317 37467254 PMC10389745

[71] Early wave of flu brings early flu hospitalizations. 2022. https://www.cdc.gov/flu/spotlights/2022-2023/early-wave-hospitalizations.htm

[72] Influenza Activity in the United States during the 2022–2023 Season and Composition of the 2023–2024 Influenza Vaccine. Centers for Disease Control and Prevention. 2023. https://www.cdc.gov/flu/whats-new/22-23-summary-technical-report.html

[73] Centers for Disease Control and Prevention. Influenza Activity in the United States during the 2023–2024 Season and Composition of the 2024–2025 Influenza Vaccine. 2024. https://www.cdc.gov/flu/whats-new/22-23-summary-technical-report.html

[74] Nielsen BF, Saad-Roy CM, Metcalf CJE, Viboud C, Grenfell BT. Eco-evolutionary dynamics of pathogen immune-escape: deriving a population-level phylodynamic curve. bioRxiv. 2024. 2024–07. 10.1098/rsif.2024.0675 PMC11963905 40172571

[75] Bull JJ, Koelle K, Antia R. Waning immunity drives respiratory virus evolution and reinfection. bioRxiv. 2024. 2024–07. 10.1093/emph/eoaf002 PMC12121555 40443498

