# Supplementary material for: Shifts in seasonal timing of respiratory diseases and causes of death following a natural pandemic event
Source: PLOS Glob Public Health. 2026 Jul 15;6(7):e0006376. doi: 10.1371/journal.pgph.0006376 (PMC13372167; doi:10.1371/journal.pgph.0006376)
Supplement: S1 Fig — (PDF) [file pgph.0006376.s001.pdf]

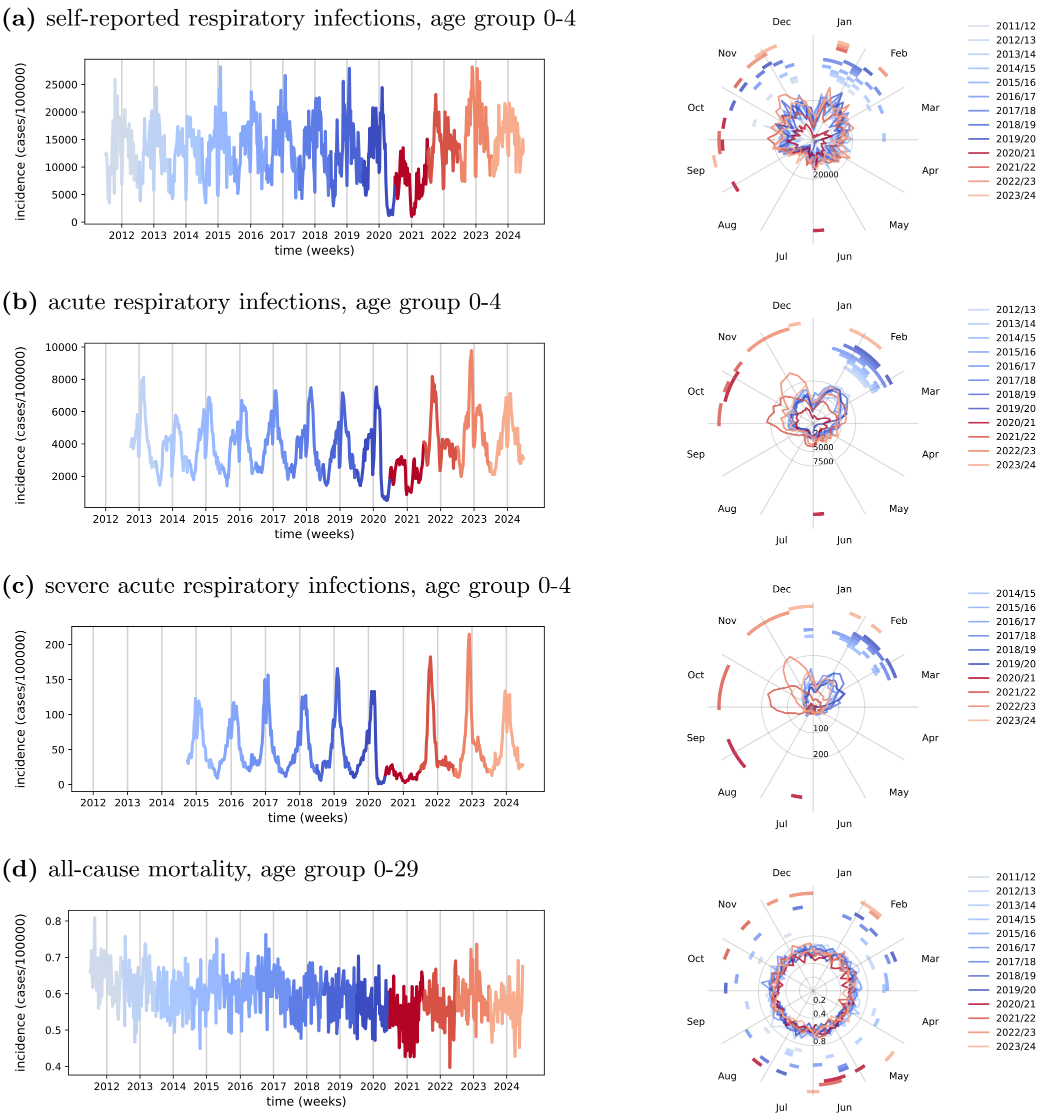

S1 Fig. Weekly incidences of respiratory infections in Germany, age group 0-4. Timeseries on the left and same data in a polar plot on the right to show the shift in peak respiratory infection season. a Self-reported, symptomatic respiratory infections (SRI). b Acute respiratory infections (ARI). c Hospitalized severe acute respiratory infections (SARI). d All-cause mortality as number of deaths per 100.000 per week.
