# Supplementary material for: Shifts in seasonal timing of respiratory diseases and causes of death following a natural pandemic event
Source: PLOS Glob Public Health. 2026 Jul 15;6(7):e0006376. doi: 10.1371/journal.pgph.0006376 (PMC13372167; doi:10.1371/journal.pgph.0006376)
Supplement: S4 Fig — (PDF) [file pgph.0006376.s004.pdf]

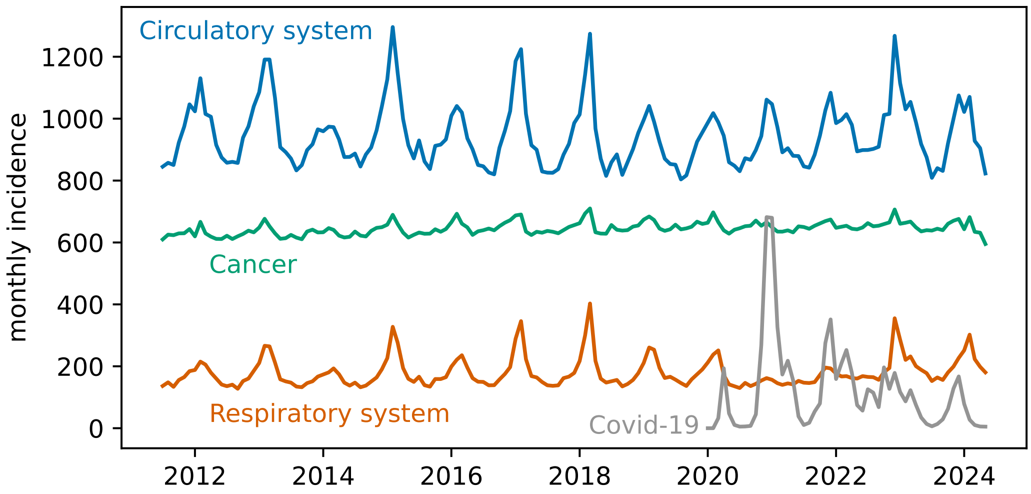


**S4 Fig.** Timeseries of monthly incidences of major causes of death in Germany. Mortality attributed to diseases of the circulatory system shows a clear seasonal pattern.
