# Supplementary material for: Shifts in seasonal timing of respiratory diseases and causes of death following a natural pandemic event
Source: PLOS Glob Public Health. 2026 Jul 15;6(7):e0006376. doi: 10.1371/journal.pgph.0006376 (PMC13372167; doi:10.1371/journal.pgph.0006376)
Supplement: S5 Fig — (PDF) [file pgph.0006376.s005.pdf]

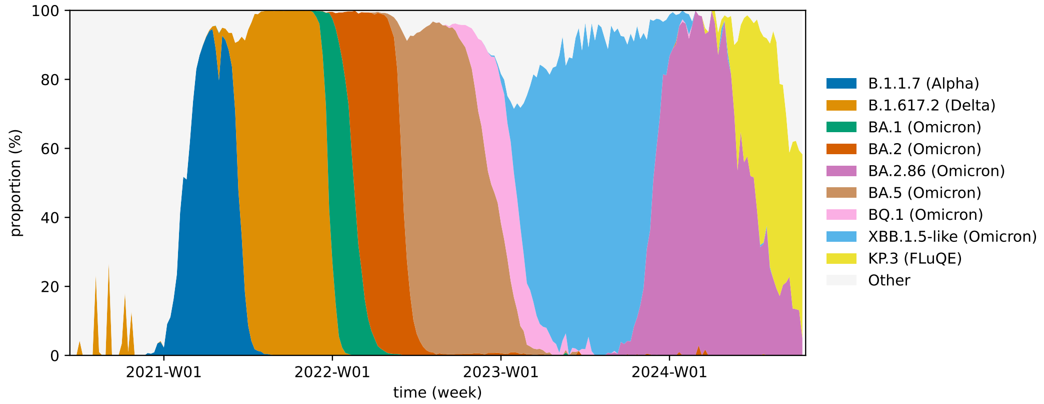

S5 Fig. Timeline of major SARS-CoV-2 variants in Germany. Shown are only variants which reached a proportion of at least 25% at some point. Data source: European Centre for Disease Prevention and Control, `http://github.com/EU-ECDC/Respiratory_viruses_weekly_data`.
