# Supplementary material for: Shifts in seasonal timing of respiratory diseases and causes of death following a natural pandemic event
Source: PLOS Glob Public Health. 2026 Jul 15;6(7):e0006376. doi: 10.1371/journal.pgph.0006376 (PMC13372167; doi:10.1371/journal.pgph.0006376)
Supplement: S6 Fig — (PDF) [file pgph.0006376.s006.pdf]

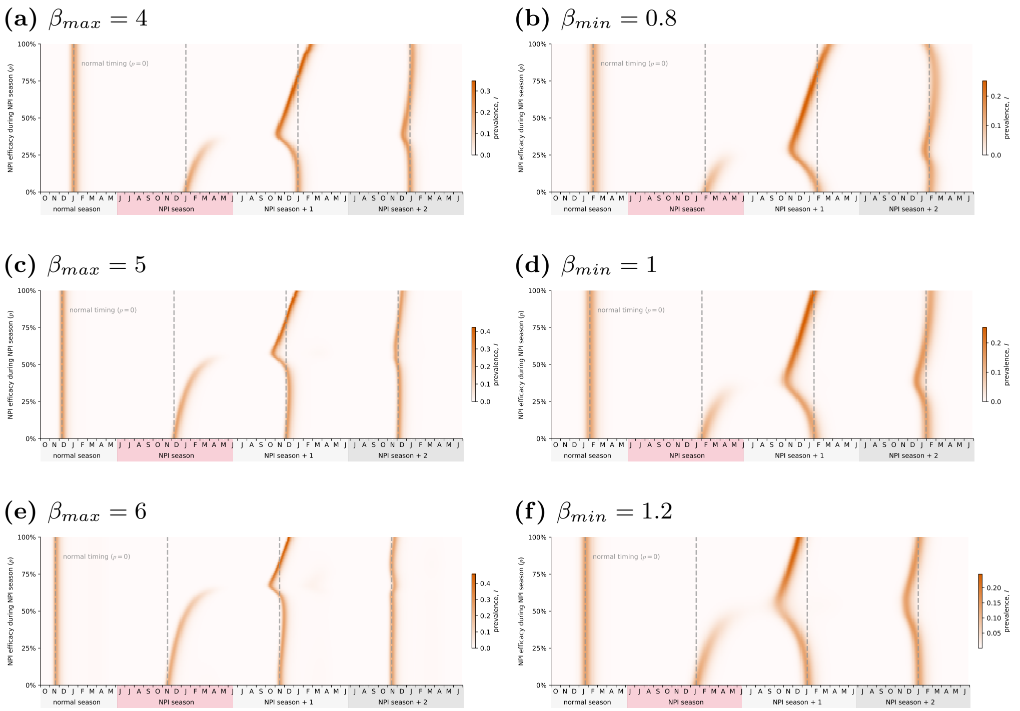

S6 Fig. (a),(c),(e) In the absence of NPIs ($p = 0$), increasing the maximum transmission rate β_{max} moves the seasonal peaks to an earlier timepoint, from late February for $\beta_{max} = 3$ to November for $\beta_{max} = 6$. An NPI season causes a similar disruption as described in the main text for $\beta_{max} = 3$, but the effect is diminished at higher transmission rates as the usual seasonal peak gets closer to its earliest possible timing. (b),(d),(f) Increasing the minimum transmission rate β_{min} also overall moves the seasonal peak forward, and exacerbates the effect of an NPI season.
