# Supplementary material for: Shifts in seasonal timing of respiratory diseases and causes of death following a natural pandemic event
Source: PLOS Glob Public Health. 2026 Jul 15;6(7):e0006376. doi: 10.1371/journal.pgph.0006376 (PMC13372167; doi:10.1371/journal.pgph.0006376)
Supplement: S7 Fig — (PDF) [file pgph.0006376.s007.pdf]

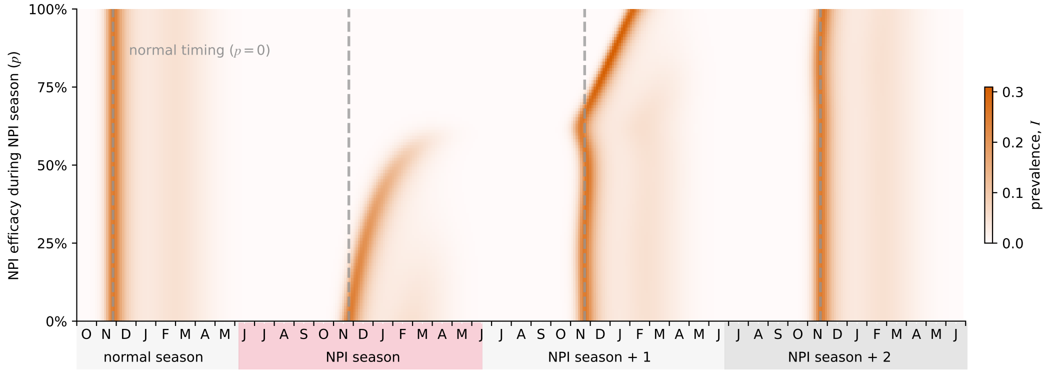

S7 Fig. Duration of immunity $\omega^{-1} = 10$ weeks. If immune waning happens much faster than the period of the underlying seasonal forcing (52 weeks), buildup of susceptibles is so fast that disruptions of the usual cycle have little effect, other than delaying the next season at very high NPI efficacies due to the disease being almost driven to extinction.
